# Artificial intelligence and skin cancer

**DOI:** 10.3389/fmed.2024.1331895

**Published:** 2024-03-19

**Authors:** Maria L. Wei, Mikio Tada, Alexandra So, Rodrigo Torres

**Affiliations:** ^1^Department of Dermatology, University of California, San Francisco, San Francisco, CA, United States; ^2^Dermatology Service, San Francisco VA Health Care System, San Francisco, CA, United States; ^3^Institute for Neurodegenerative Diseases, University of California, San Francisco, San Francisco, CA, United States; ^4^School of Medicine, University of California, San Francisco, San Francisco, CA, United States

**Keywords:** artificial intelligence, skin cancer, melanoma, dermatology, dermatopathology

## Abstract

Artificial intelligence is poised to rapidly reshape many fields, including that of skin cancer screening and diagnosis, both as a disruptive and assistive technology. Together with the collection and availability of large medical data sets, artificial intelligence will become a powerful tool that can be leveraged by physicians in their diagnoses and treatment plans for patients. This comprehensive review focuses on current progress toward AI applications for patients, primary care providers, dermatologists, and dermatopathologists, explores the diverse applications of image and molecular processing for skin cancer, and highlights AI’s potential for patient self-screening and improving diagnostic accuracy for non-dermatologists. We additionally delve into the challenges and barriers to clinical implementation, paths forward for implementation and areas of active research.

## Introduction

Artificial intelligence (AI) stands at the forefront of technological innovation and has permeated into almost every industry and field. In dermatology, significant progress has been made toward the application of AI in skin cancer screening and diagnosis. Notably, a milestone that marked the era of modern artificial intelligence in dermatology was the demonstration of skin cancer classification abilities by deep learning convolutional neural networks (CNNs), which was on par with the performance of board-certified dermatologists (1). This CNN was trained on a dataset that was two orders of magnitude greater than those previously utilized. The dermatologist-level classification ability has since been experimentally validated by other papers (2, 3). Recent progress in the field of AI enables models to not only analyze image data but also integrate clinical information, including patient demographics and past medical history (4–6). Advancements allow for the simultaneous evaluation and identification of multiple lesions from wide-field images (7, 8). Moreover, models can now gain information from whole slide images without having to use costly pixel-wise human-made annotations (9). Despite these advancements, research has found that AI models lack robustness to simple data variations, have proven inadequate in real-world dermatologic practice performance, and that barriers remain before achieving clinical readiness (2, 10–14).

## Clinical applications

Artificial intelligence has been employed to predict the most common types of skin cancers, melanoma (1) and non-melanoma skin cancer (1), through image analysis. In addition, machine learning has been used on RNA datasets to develop classifiers that also predict skin cancer, as well as the prognosis of skin lesions. Several of these methods can be, or have the potential to be, readily deployed by patients, primary care practitioners, dermatologists, and dermatopathologists.

### Patients

With the rising prevalence of smartphone usage, patients can directly screen for and monitor lesions with AI applications. These applications can run AI models on patients’ own local devices, which ensures the protection of patient data (15). The feasibility of an AI model to assist patients’ with self-assessed risk using smartphones has been validated with a model that was trained on pictures captured from patients’ smartphones, and which exhibited comparable performance to general practitioners’ ability to distinguish lower-risk vs. higher-risk pigmented lesions (16). Moreover, AI significantly increased the abilities of 23 non-medical professionals to correctly determine a diagnosis of malignancy from 47.6 to 87.5% without compromising specificity (12). In the future, AI models may assist with overseeing and assessing changes to lesions as they progress (17) and collaborate with apps that allow patients to examine themselves and document moles (18, 19).

Despite progress with these AI models, there is no smartphone application that is endorsed on the market in the United States for non-professionals to evaluate their lesions as they do not have satisfactory performance or generalizability (20). Limitations include biases introduced due to the narrow range of lesion types, skin pigmentation types, and low number of high-quality curated images used in training. Further, inadequate follow-up has been a limitation with regards to identifying false negative diagnoses (21). Notably, users may not be adequately protected from the risks of using smartphone diagnostic apps by Conformit Europenne (CE) certification, which endorsed two apps with flaws (SkinVision and TeleSkin’s skinScan app). A prospective trial of SkinVision found low sensitivity and specificity for melanoma classification (22). In contrast to CE, the US Food and Drug Administration’s (FDA) requirements for endorsement are more stringent (21).

### Primary care

Artificial intelligence applications can enhance skin cancer screening in the primary care setting and streamline referrals to dermatologists. Referral data from primary care practitioners to teledermatology consultations were used to train a model capable of a top-3 accuracy and specificity of 93 and 83%, respectively, given 26 skin conditions that makeup 80% of encountered primary care cases (4). This performance was on par with dermatologists and surpassed primary care physicians (PCPs) and nurse practitioners. This type of model could assist PCPs in diagnosing patients more accurately and broadening their differential diagnoses. In cases in which the top 3 diagnoses from the model have the same management strategy, patients may start treatment while awaiting further workup or follow-up with dermatology. Nevertheless, further testing on populations with a low prevalence of skin cancer is essential to demonstrate efficacy in the broader population (23).

### Dermatology

Models have been trained to use electronic health record (EHR) data and/or gene sequencing data to predict an individual’s likelihood of developing melanoma (24–27) or nonmelanoma skin cancer (27–31). While AI models could potentially flag patients at high risk of skin cancer to be screened, studies are limited by the variability of included predictive factors, inconsistent methods of evaluating models, and inadequate validation (32). Moreover, EHRs often do not include some of the most important risk determinants for skin cancer, such as exposure to UV light and the patient’s familial history; the omission of such data may result in decreased performance (28).

Artificial intelligence has the potential to supplement dermatologists’ diagnostic and treatment capabilities in what is known as augmented intelligence (AuI). For diagnosis, AuI might assist dermatologists in more effectively managing teledermatology referrals (4) and increase the efficacy of in-person visits (33). However, in a prospective trial comparing AI to dermatologists in a teledermatology setting, dermatologists outperformed the AI (13). Despite AI currently underperforming dermatologists, AI could provide a new perspective that could still be beneficial as AI and humans exhibit distinct types of errors. For instance, models may provide insights into certain images’ classification ambiguity, whereas humans are better able to distinguish variability in image quality such as blurriness or shadowing (12).

Augmented intelligence can also assist with suggesting clinical decisions given inputted images, such as recommending whether a lesion warrants excision (34). The integration of AuI into dermatologic patient management resulted in a 19.2% reduction in unnecessary excisions of benign lesions (35). Although current CNNs’ performance has been shown to fall short when compared with using sequential dermatoscopic photography in predicting melanoma, AuI may be used in the future by dermatologists to evaluate and monitor lesion change (36). Of interest, in this study, neither dermatologists nor the CNN had satisfactory diagnostic performance levels on baseline images, but both dermatologists and CNN had improved performances when follow-up images were provided, and the best performance was combining CNN and dermatologist assessment together.

Integration of AI into advanced imaging techniques may reduce the extent of training necessary to use them (37). One area of application is in the detection of the dermal-epidermal junction, which is crucial in a non-invasive method of skin cancer diagnosis called reflectance confocal microscopy (RCM) imaging (38). Furthermore, there are ongoing efforts to analyze RCM images with AI (39).

The FDA has not approved any medical devices or algorithms based on artificial intelligence in the field of dermatology (40, 41). On the other hand, the FotoFinder Moleanalyzer Pro, an AI application for dermatology, was approved in the European market. It demonstrated performance on par with dermatologists in store-and-forward dermatology (42) and a prospective diagnostic study (43), however, the latter had extensive exclusion criteria, e.g., excluding patients of skin type IV and greater. The first randomized controlled trial comparing AI skin lesion prediction to dermatologists’ assessment reported that AI did not exceed attending dermatologists in skin cancer detection (44).

### Dermatopathology

With the growing application of whole slide imaging (WSI) in the field of dermatopathology (45), AI can potentially support dermatopathologists in several ways, particularly skin cancer recognition. Among the AI models trained to detect melanoma from digitized slides (5, 46–50), two models were able to match the performance of pathologists in an experimental setting. These models were limited in that they were only given either a part of (46) or a single (49) hematoxylin and eosin (H&E)-stained slide. In contrast, pathologists can utilize supplementary data such as immunohistochemistry or relevant patient data. However, integrating patient information, such as age, sex, and lesion location, into CNN models did not enhance performance (5). One limitation to implementing AI in dermatopathology is the unreliable prediction that may be made when a model is given an input that differs from the training dataset. One potential solution is the use of conformal prediction, which has been shown to increase accuracy of prostate biopsy diagnosis by flagging unreliable predictions (51).

Studies have been done to evaluate AI’s ability for diagnosing basal cell carcinoma (BCC) using WSI (9, 52, 53). Campanella et al. showed the ability of a convolutional neural network to achieve 100% sensitivity for detecting BCC, on the test set; importantly, a multiple instance learning approach was introduced that obviated the necessity of time-consuming pixel-level slide annotations to distinguish between areas with and without disease (9). Kimeswenger et al. subsequently incorporated an “attention” function to draw attention to areas of digital slides that include indications of BCC. Interestingly, CNN pattern recognition varied from that of pathologists for BCC diagnosis as tissues were flagged based on different image regions (53). These CNNs could also be applied to identify and filter slides for Mohs micrographic surgery (52). In the setting of rising caseloads, AI can help to decrease pathologists’ workload generated by these commonly diagnosed, low risk entities. Duschner et al. applied AI to automated diagnosis of BCCs, and demonstrated both sensitivity and specificity of over 98%. Notably, the model demonstrated successful generalization to samples from other centers with similar sensitivity and specificity (54).

Artificial intelligence has also had some success in predicting sentinel lymph node status (55), visceral recurrence, and death (56) based on histology of primary melanoma tumors. In the future, AI could be utilized to identify mitotic figures, delineate tumor margins, and determine the results of immunohistochemistry stains; further, AI could recommend more immunostaining or genetic panels that could be of use diagnostically (57). While AI predictions have not been consistently successful for melanoma (58), AI has been demonstrated to identify the mutation given a lung adenocarcinoma slide that has been stained with H&E (59–61).

### Machine learning applied to RNA profiles

While AI in dermatology is most often associated with using deep learning techniques on clinical and histological images, machine learning methods have been utilized in developing gene expression profile (GEP) classifiers for predicting skin cancer diagnosis and prognosis. Generally, simpler machine learning models that require tuning of fewer parameters compared to more complex neural nets have been employed to analyze GEP. They still, however, share the benefits of the ability to use iterative learning optimized to find patterns in complex non-linear relationships not possible in traditional statistical and linear models, assuming sufficient data is available. Some common models include many Kernel methods such as support vector machines (SVM) or tree-based models, e.g., Random Forest and XGBoost that have often been found to produce the best performance for tabular gene expression data. These models also often use some method to feature select (62) to both maximize performance and find the most relevant features for the classification task. This also allows for a better sense of interpretability as with fewer features there is the ability to assess their relevance individually. Reproducibility is of great concern and has often been the critique of many biomarker and classifier studies, since there is often little to no overlap in targets, which understandably can lead to general skepticism of the results, especially considering the generally small sample sizes employed in many studies. Despite this, there has been a push to make use of molecular profiling to assist in different aspects of melanoma management.

Currently, the GEPs developed for use in melanoma management fall into two categories. First, some GEPs are used as a diagnostic tool to help determine the malignancy of a pigmented lesion either pre- or post-biopsy. Pre-biopsy there is an epidermal tape sampling test that can predict melanoma with 94% sensitivity and 69% specificity (63) with an improved sensitivity of 97%, when TERT mutation assessment is included (64). There are, however, reported limitations to this test as it cannot be used on mucous membranes or acral skin and there is the possibility of non-actionable results due to insufficient sample collected for testing (65). Post-biopsy GEPs can be used to help with diagnostically difficult cases such as Spitz nevi, but have poorer performance on Spitz melanomas and pediatric patients (66). Machine learning has also been applied with success to miRNA profiles to differentiate melanomas from nevi (67).

Second, there are GEPs, derived from biopsy material, that are used as prognostic tools to stratify the risk of melanoma recurrence or metastasis (68), however subsequent management protocols for high risk early-stage disease are not in place (68). Despite optimism for prognostic use of prognostic GEP classifiers, the expert consensus is that there is currently insufficient evidence to support routine use (69). The climate, however, is evolving, with new reports incorporating additional clinicopathological data together with patient outcomes (70). Overall, there remains a lack of consensus on the use of the GEP biopsy and tape sampling tests (71, 72). Further studies are needed, such as non-interventional retrospective studies, followed by prospective interventional trials, but there remains promise that they can become additional tools in providers’ arsenal of available tests.

## Barriers to clinical implementation

### Image quality

Image quality significantly impacts the prediction performance of AI computer vision (73). Several factors can result in subpar images, including inadequate focus or lighting, color misrepresentations, unfavorable angles or framing, obstructing objects, and poor resolution. Moreover, while humans can readily ignore items such as blurred focus, scale bars, and surgical markings, these artifacts affect AI prediction performance (11, 74, 75).

Obtaining consistently high-quality images in the fast-paced environment of a clinic presents many challenges. Barriers such as limited time, insufficient training, inadequate imaging equipment, and other constraints may hinder the process. Guidelines for skin lesion imaging have been suggested to facilitate the capture of high-quality images (76, 77). These guidelines include suggestions for adequate lighting, background, field of view, image orientation, and color calibration. Additional recommendations are suggested for photographing skin of color (78).

A comprehensive, multifaceted solution is necessary to enhance image quality. Educating dermatology residents in photography might contribute to improving image quality in a clinical setting (79). Moreover, a study done in United Kingdom primary care facilities showed enhanced photo quality when patients were educated with the “4 Key Instructions” (Framing—requesting at least one near and one distant image; Flash—educating about the use of flash to enhance image sharpness, emphasizing not to use it too closely; Focus—educating patients to give the camera time to auto-focus; Scale—asking for a comparison like a ruler or a coin) (73). Among 191 digital applications for skin imaging, 57% included one or more strategies to enhance quality, but it was rare for applications to have more than one (80). An immediate feedback feature for image quality shows promise, although it is still in the early stages of development (81).

### Algorithmic bias and health equity

There is a risk for indiscriminately implemented AI to potentially exacerbate health inequities by incorporating pre-existing and newly emerging biases (82) (Table 1). Pre-existing biases include pre-coding biases in datasets used to train the model or personal biases inadvertently introduced by developers. Emergent biases can be introduced by relying on models in new or unexpected contexts and not adjusting models for new knowledge and shifting cultural norms.

**Table 1:** Challenges in AI in dermatology.

Challenges	Summary
Model validation	Many models fail to have a true external validation set so can fail to be representative of the general population. In addition, standardized benchmarks that can be used across models are not readily available due to limitations with few public datasets that serve as good benchmarks.
Quality of data	Model performance can be limited by quality of data, which can be affected at initial collection through user error creating data artifacts or with intrinsic deficiencies of the source limiting diversity and creating class imbalances that are not accounted for by the model.
Algorithmic bias and health equity	Models can contain biases based on the selection of data used to train that can affect generalizability to different demographics both racial and socioeconomic.
Implementation and user confidence	Acceptance of AI can be limited not only by governmental agencies such as FDA approving use, but also at the clinician and patient level where mistrust or uncertainty can dissuade use.

Artificial intelligence models for early melanoma detection have relied on large datasets from individuals with mostly lighter pigmented skin. While melanoma is more prevalent among individuals with lighter skin, those with darker skin frequently come in with a more severe stage of disease and experience lower survival rates. An AI model trained on lighter skin tones for melanoma prediction had lower performance for lesions on darker skin tones (83). The International Skin Imaging Collaboration (ISIC) archive, one of the most extensive and widely used databases for individuals in the United States, Europe, and Australia, and a prospective diagnostic accuracy paper comparing an AI model with other noninvasive imaging techniques did not include individuals with Fitzpatrick phototype III or higher (43, 84). Efforts to collect lesions from individuals of all skin tones should be a priority, and transparency in the characteristics of training datasets as well as the quality and range of disease labels should be disclosed (85).

### AI model validation

It is crucial to carefully validate AI models before applying them in real-world settings (Table 1). Computational stress testing is necessary to guarantee efficacy in actual clinical scenarios (2). Validation should be performed using large amounts of external data as determining performance solely on internal data has been shown to often lead to overestimation (2, 86). The reason for the lower model performance on external validation datasets can arise from training data that is not representative of the general population or from leakage of additional data, either between the training and testing data or from the future drift of data (86). Unfortunately, most models are not open code, limiting research into the external validation of these models. On the other hand, Han et al. share the use of their models publicly, setting a standard that should be followed (7, 12, 87). Along with publicly shared models, having publicly shared benchmarks such as the melanoma classification benchmark (88) and accessible databases (such as DataDerm) is crucial for comprehensive validation (89). Few public datasets have representation of all skin types. A rigorous testing of outcome metrics with and without the support of an AI model in randomized controlled trials would be optimal.

Though CNNs routinely and autonomously identify image features pertinent for classification, this ability can lead to the incorporation of unintended biases. An example of possible bias is the use of ink markings (75) or scale bars (74) in melanoma identification. It is important to assess whether and how changes to inputted images can affect the prediction output. Changes to test include image quality, rotation, brightness/contrast adjustments, adversarial noise, and the presence of artifacts, such as those aforementioned (2, 10, 74, 75, 90, 91). Testing for robustness given such uncertainties can assist users in understanding the model’s scope and reasons for error (92).

## The path to clinical implementation

Given the rapid pace of advancements in AI in the medical field, the American Academy of Dermatology (AAD) issued a position statement regarding how to integrate augmented intelligence into dermatologic clinical settings (93). The AAD underscored the importance of high-quality validated models, open transparency to patients and providers, and efforts to actively engage stakeholders.

For AI to be broadly accepted in dermatology, studies need to demonstrate a significant improvement in health outcomes. The first randomized controlled trial of an AI’s ability to augment clinicians’ diagnostic accuracy on skin lesions highlighted the potential for AI to augment non-dermatologists diagnostic performance in a real-world setting, but not that of dermatology residents in training, and found superior performance by experienced dermatologists—who use patient metadata as well as images—compared to the AI model (44). It also noted that if the model’s top 3 diagnoses were incorrect, trainees’ diagnostic accuracy fell after consulting the AI model, highlighting a pitfall of using current AI models.

### Increasing access to dermatological care

AI offers hope for increasing health equity through increasing access, and democratizing skin screenings. Access to dermatologists is a problem, especially in rural areas, where it may take longer for a patient to obtain a biopsy of suspected melanoma (94). As of 2018, 69% of counties in the United States do not have access to dermatologists (95). Further exacerbating the issue, many dermatology clinics closed during the COVID-19 pandemic (96). AI-augmented teledermatology may be able to enhance accessibility by streamlining referrals and reducing waiting times, and it could help increase the accessibility in areas with a scarcity of dermatopathologists. AI may also help dermatologists more accurately diagnose skin disease in patients whose skin is not well-represented in the local population (97).

### Human-computer collaboration

Clinicians are indispensable to synthesizing relevant context and offering patient counseling and subsequent care. Furthermore, given the enhanced accuracy of diagnosis when integrating AI into decision-making, the future of dermatology will likely entail human-computer collaboration (98). Embedding Collective Human Intelligence (CoHI) or even swarm intelligence (CoHI with interaction between participating humans) as checkpoints within an AI model may help overcome the limited ability of AI to contextualize and generalize (99).

When interacting with AI, potential cognitive errors and biases may be exacerbated, especially when there is discordance in diagnosis between clinicians and AI (100). The use of AI introduces a new kind of bias called automation bias, in which humans tend to unquestioningly trust automated decisions from AI (100). When physicians used AI decision support for reading chest X-rays, experienced physicians rated diagnostic advice as lower quality when they thought the advice was generated by AI, but not physicians with less experience (101). Though rated as less trustworthy, inaccurate advice by AI still led to decreased diagnostic accuracy (101). It will be important for AI developers and medical educators, the latter when teaching AI applications, to take such human factors into account.

## Areas of active research

There are several areas of active computational research that are anticipated to aid in bringing validated image analysis models to clinical use (Table 2).

**Table 2:** Future advances in AI.

Method	Description
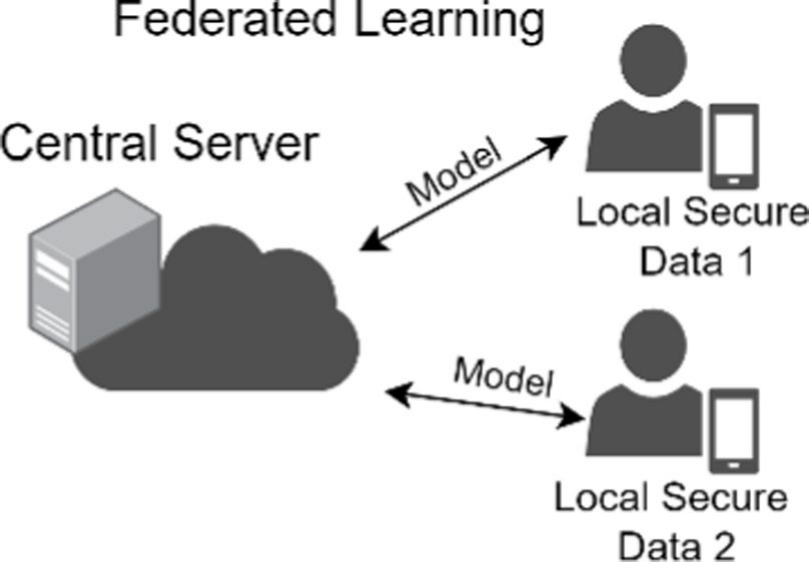	Uses decentralized training where a global model is trained on locally-stored data and then updated while preserving the privacy of local data.
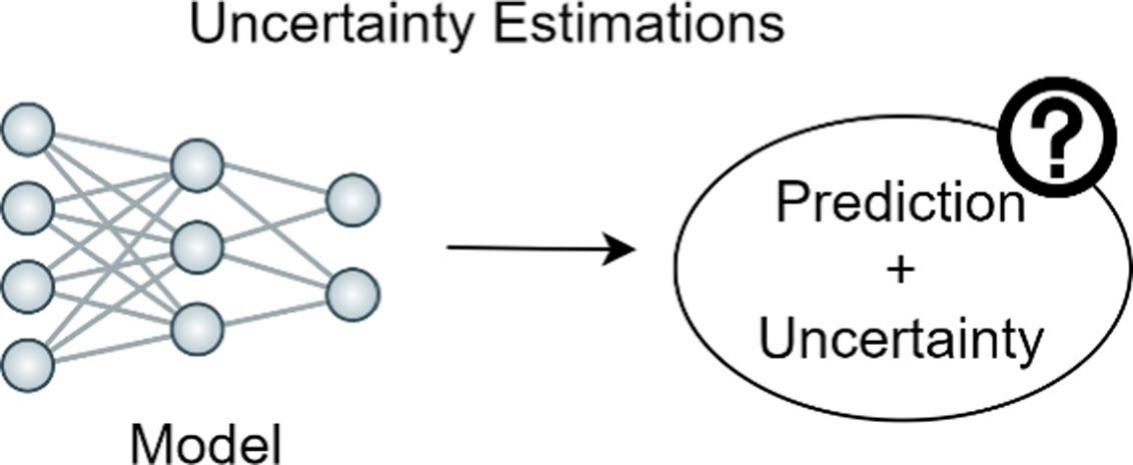	To calculate an uncertainty estimate for model predictions so model confidence can be interpreted by end user.
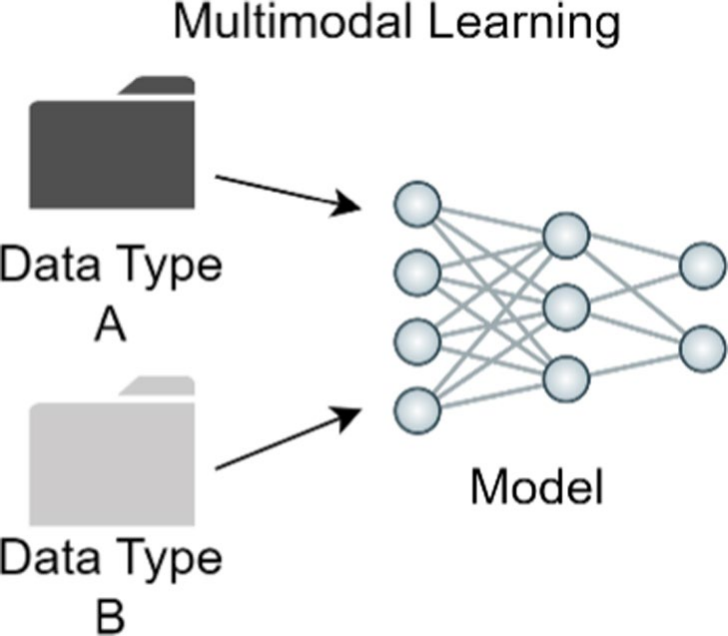	Allows the use of multiple types of data to train a combined model to take advantage of unique differences in data.
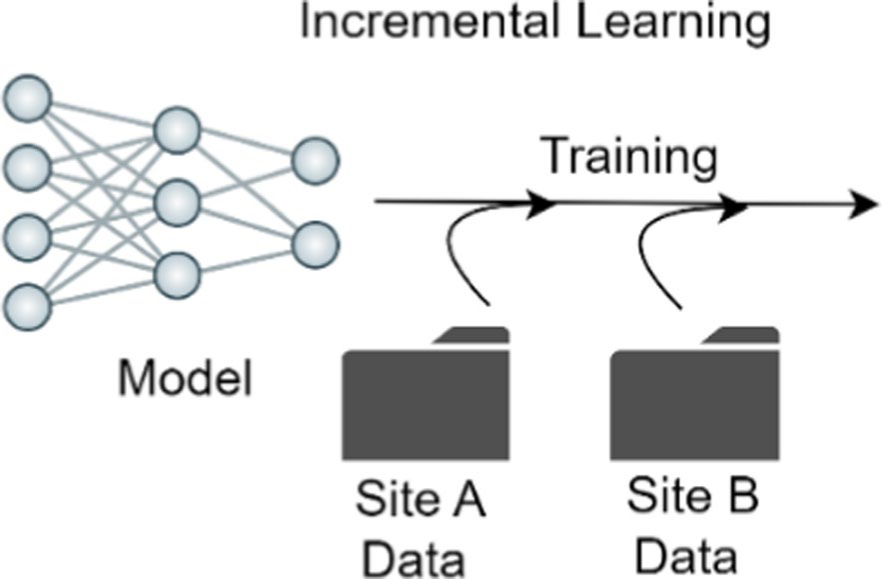	Enables a model to continue learning on a new stream of data.
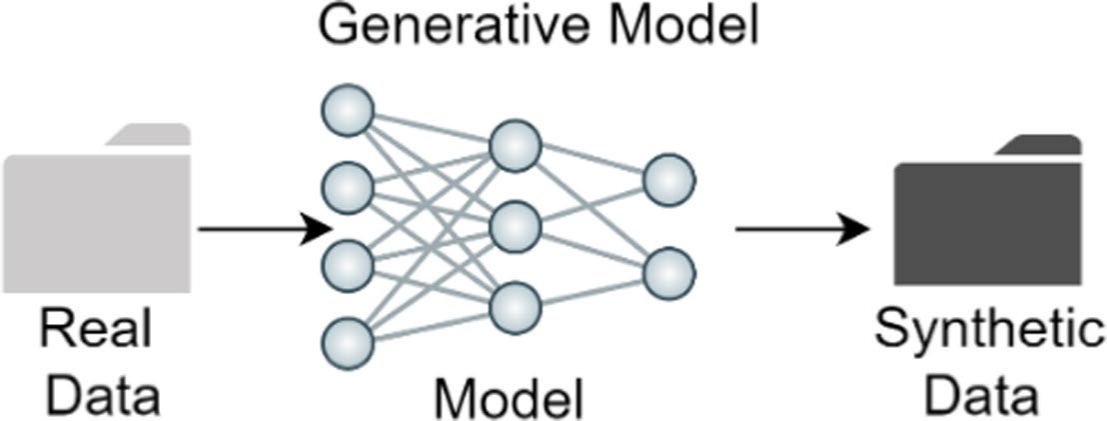	Model is used to train compressed representations of data so new instances can be recreated.
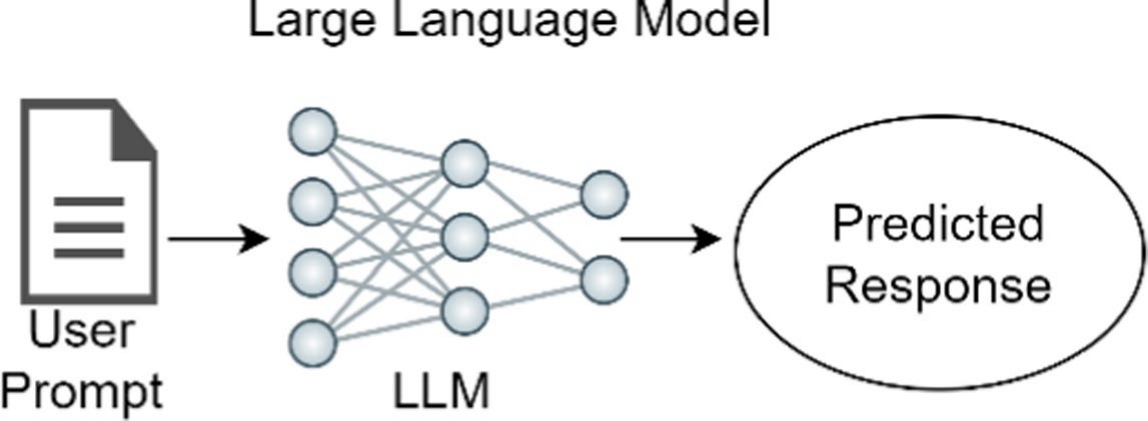	Model uses text based prompt to generate a response based on language based learning.

### Federated learning

A problem with training models for clinical use to detect skin cancer or other disorders is the limitation in sharing clinical images due to privacy concerns and the inherent limitations in collecting sufficient images of rare skin cancer types and disorders and of different skin pigmentation. The current approach for multi-institution model training necessitates the forwarding of patient data to a centralized location, termed collective data sharing (102). Alternatively, federated learning uses a decentralized training system in which a shared global model learns collaboratively while keeping data locally. Each device’s data comes with its own inherent bias and different properties due to demographic variations. Instead of sending data to a central server, the model itself travels to each device, learns from the locally-stored data, and then updates the global model with this newly acquired training. By not sharing the training data across devices, federated learning enables the preservation of privacy of sensitive data (103). In a study across 10 institutions, the performance from federated learning was shown to better than that of a single institution model and shown to be comparable to that of collective data sharing (102). Moreover, the federated learning approach would be a method to virtually aggregate data on rare skin cancers or disorders from different centers, such as Merkel cell cancer, or data from patients with rarer subtypes of skin cancers, such as mucosal or acral melanoma. An analogy of federated learning is a team of dermatologists who visit multiple clinics to learn and share knowledge, rather than asking patients to visit a single central hospital to see the team. A model trained with federated learning can offer more accurate diagnoses on rare skin cancer types and disorders, including lesions found on differing skin pigmentations, and still maintain patient privacy.

Deploying federated learning faces several challenges. Ensuring fairness across different demographic groups and data security while optimizing the overall performance of the global model is computationally complicated. Establishing computational infrastructure capable of seamless communication, such as transmission of a model, may require additional IT assistance. These obstacles pose a barrier to the practical implementation of federated learning (104).

### Uncertainty estimation

Whereas many studies on the applications of skin cancer classification models have reported high accuracy, these models rarely concurrently report uncertainty estimates for the predictions and when assessed, models have been found to be overconfident (2). As a result, medical practitioners may hesitate to incorporate these models into their diagnostic workflow. Uncertainty estimation provides a meaningful confidence level, with regards to when to trust a model prediction. To safely deploy a computer-aided diagnostic system in a clinical setting, it is crucial to incorporate not only a model’s prediction but also a confidence score. Clinicians are then equipped to decide whether to trust the prediction or alternatively disregard the AI prediction and rely on provider assessment (94).

### Multimodal learning

Most skin disease diagnosis models are trained only on one data modality: clinical or histological images or RNA sequence data. However, medical data is inherently multimodal by nature, and dermatologists use patient information in addition to clinical images to make a diagnosis. Metadata from patients, such as age, ethnicity, and anatomic location of lesion, can also be useful to enhance skin cancer classification models. Multimodal learning is a technique where a single model learns from multiple types of data simultaneously (105). One skin disease classifier that integrated up to six clinical images and 45 demographic items and medical history to classify 26 skin conditions as the primary prediction outperformed six primary care physicians and six nurse practitioners (4). Another study showed that a model integrating dermatoscopic and macroscopic images with three patient metadata variables outperformed models with just one image modality for binary and multiclass classification setting (106, 107).

### Incremental learning

Current skin disease diagnostic models are static, wherein data distribution is already known and the target skin diseases are pre-set. However, in the clinical setting, as the database size grows over time, with the accumulation of new images, a shift in data distribution can occur, for example after the inclusion of new skin disease classes, or with improved or new devices. Changes or differences in image acquisition tools, such as mobile phone cameras, also can shift dataset distribution by changing the quality of images captured. This results in the need to adapt models to new images while not degrading model performance on the pre-existing data. Incremental learning enables a model to continue learning the attributions of new data while preserving learned features from the data acquired before; successful incremental learning strategies on dermatology images have been recently reported (97, 108, 109).

### Generative adversarial networks modeling

The ability to synthesize new data that closely resembles real skin lesion images can augment training on rare skin diseases and create a diverse and balanced dataset (110). While the potential to fill the data gaps is promising, models’ performance does not show significant improvement when trained on synthesized data (111). The stylized images should be used cautiously, so as to not degrade the quality or reliability of the dataset and model by adding unintentional bias, and also ensure alignment with real-world conditions for clinical application (111, 112).

### Emerging new model architectures—vision transformers

Vision transformer has emerged as an advanced model architecture, challenging the traditional dominance of convolutional neural networks (CNNs). CNNs have been the default choice for in both medical imaging and natural image tasks (113, 114). However, inspired by the success of Transformer in natural language processing (NLP), researchers have increasingly utilized ViTs or hybrid models of CNN and ViT and demonstrated promising results across various medical imaging tasks (115, 116). Concurrently, a resurgence of CNN is occurring with advanced CNN architectures such as ConveNeXt, showcasing competitive performance alongside Transformers in natural image task (117). These ongoing explorations and adaptations of ViTs address the challenges and uncertainties in deciding on model architecture.

### Applications of large language model

Large language model is a type of natural language processing model that is trained to “understand” and generate human-like text, and has potential applications in enhancing clinical decision-making and overall patient care. For example, ChatGPT-style LLMs designed only for clinical diagnosis can accelerate clinical diagnoses by helping patients better understand their medical conditions and communicate with doctors remotely (118). Another application of LLM in clinic is AI-enabled digital scribes that can record and summarize patients visit information for treatment plans and billing purposes, eliminating the workload due to medical charting (119, 120). While there are positive aspects of LLM utilization for clinical care, there are also concerns such as the need for continued oversight of such models. It is essential to recognize that LLMs and doctors can complement each other, with LLM providing efficiency in processing large amounts of information while doctors offer interpretation of the data, emotional intelligence and compassion to patients, thus improving patient care (121). However, caution should be used when utilizing LLM for medical advice. A recent study demonstrated that 4 LLM provided erroneous race-based responses to queries designed to detect race-based medical misapprehensions (122). To address this, testing of LLMs is critical before clinical implementation, and human feedback can help to correct errors.

### Self-supervised learning

Self-supervised learning offers a promising approach to enhance the robustness and generalizability of models by enabling them to learn meaningful representations from unlabeled data. Traditionally, the efficacy of training deep learning models has relied on access to large-scale labeled datasets (123). However, in the medical field, acquiring such data is costly and requires specialized expertise. As a result, the scarcity of annotated data poses a significant obstacle to the development of robust models for various clinical settings. SSL addresses this challenge by developing a versatile model capable of efficiently adapting to new data distributions with a reduced number of labeled data during fine-tuning, while ensuring strong performance (124). Thus, SSL is a promising method to bridge the gap between AI research in the medical field and its clinical implementation.

## Conclusion

Artificial intelligence currently is able to augment non-dermatologists’ performance in a synergistic fashion and performs at the level of experienced dermatologists in a randomized controlled trial assessing skin malignancies. This achievement opens the door to aiding primary care physicians’ discriminative triaging of patients to dermatologists and likely will decrease referrals for benign lesions, thereby freeing up dermatology practices to address true malignancies in a timely manner. Similarly, the potential for patients to self-refer for lesions concerning for malignancy may be possible in the near future, with models that can assess regional anatomic sites for lesions with concerning features. Through the implementation of AI, access to dermatologic care may become more democratic and accessible to the general population, including underserved subpopulations.

Limitations in performance include misdiagnosis by the model when assessing out of distribution diagnoses, leading clinicians astray; a solution might be for models to provide confidence estimates together with diagnostic predictions. A formidable problem in training models is the large number of diagnoses in dermatology, including numerous low incidence but aggressive malignancies (such a Merkel cell carcinoma, microcytic adnexal carcinoma, dermatofibrosarcoma tuberans, and angiosarcoma), or low incidence chronic malignancies such as cutaneous T cell lymphoma with potential for aggressive progression; one solution is federated training through the collaboration of multiple academic centers, some of which have specialty clinics focused on these diagnoses; or the formation of a central shared databank. In the future, models likely will be utilized to aid experienced dermatologists and dermatopathologists, as well as primary care providers and patients, particularly after training on multimodal datasets.

## Author contributions

MW: Writing – review & editing, Writing – original draft, Supervision, Resources, Conceptualization. MT: Writing – review & editing, Writing – original draft. RT: Writing – review & editing, Writing – original draft, Visualization. AS: Writing – original draft, Writing – review & editing.

## References

[1] Esteva A Kuprel B Novoa RA Ko J Swetter SM Blau HM. Dermatologist-level classification of skin cancer with deep neural networks. Nature. (2017) 542:115–8. doi: 10.1038/nature21056 28117445 PMC8382232

[2] Young AT Fernandez K Pfau J Reddy R Cao NA von Franque MY. Stress testing reveals gaps in clinic readiness of image-based diagnostic artificial intelligence models. NPJ Digit Med. (2021) 4:10. doi: 10.1038/s41746-020-00380-6 33479460 PMC7820258

[3] Young AT Xiong M Pfau J Keiser MJ Wei ML. Artificial intelligence in dermatology: a primer. J Invest Dermatol. (2020) 140:1504–12. doi: 10.1016/j.jid.2020.02.026 32229141

[4] Liu Y Jain A Eng C Way DH Lee K Bui P. A deep learning system for differential diagnosis of skin diseases. Nat Med. (2020) 26:900–8. doi: 10.1038/s41591-020-0842-3 32424212

[5] Höhn J Krieghoff-Henning E Jutzi TB von Kalle C Utikal JS Meier F. Combining CNN-based histologic whole slide image analysis and patient data to improve skin cancer classification. Eur J Cancer. (2021) 149:94–101. doi: 10.1016/J.EJCA.2021.02.032 33838393

[6] Rotemberg V Kurtansky N Betz-Stablein B Caffery L Chousakos E Codella N. A patient-centric dataset of images and metadata for identifying melanomas using clinical context. Sci Data. (2021) 8:1–8. doi: 10.1038/s41597-021-00815-z 33510154 PMC7843971

[7] Han SS Moon IJ Lim W Suh IS Lee SY Na JI. Keratinocytic skin Cancer detection on the face using region-based convolutional neural network. JAMA Dermatol. (2020) 156:29–37. doi: 10.1001/jamadermatol.2019.3807 31799995 PMC6902187

[8] Soenksen LR Kassis T Conover ST Marti-Fuster B Birkenfeld JS Tucker-Schwartz J. Using deep learning for dermatologist-level detection of suspicious pigmented skin lesions from wide-field images. Sci Transl Med. (2021) 13:eabb3652. doi: 10.1126/SCITRANSLMED.ABB3652 33597262

[9] Campanella G Hanna MG Geneslaw L Miraflor A Werneck Krauss Silva V Busam KJ. Clinical-grade computational pathology using weakly supervised deep learning on whole slide images. Nat Med. (2019) 25:1301–9. doi: 10.1038/s41591-019-0508-1 31308507 PMC7418463

[10] Maron RC Haggenmüller S von Kalle C Utikal JS Meier F Gellrich FF. Robustness of convolutional neural networks in recognition of pigmented skin lesions. Eur J Cancer. (2021) 145:81–91. doi: 10.1016/J.EJCA.2020.11.020 33423009

[11] Maier K Zaniolo L Marques O. Image quality issues in teledermatology: a comparative analysis of artificial intelligence solutions. J Am Acad Dermatol. (2022) 87:240–2. doi: 10.1016/J.JAAD.2021.07.073 34384833

[12] Han SS Park I Eun Chang S Lim W Kim MS Park GH. Augmented intelligence dermatology: deep neural networks empower medical professionals in diagnosing skin cancer and predicting treatment options for 134 skin disorders. J Invest Dermatol. (2020) 40:1753–61. doi: 10.1016/j.jid.2020.01.019 32243882

[13] Muñoz-López C Ramírez-Cornejo C Marchetti MA Han SS Del Barrio-Díaz P Jaque A. Performance of a deep neural network in teledermatology: a single-Centre prospective diagnostic study. J Eur Acad Dermatol Venereol. (2021) 35:546–53. doi: 10.1111/JDV.16979 33037709 PMC8274350

[14] Agarwala S Mata DA Hafeez F. Accuracy of a convolutional neural network for dermatological diagnosis of tumours and skin lesions in a clinical setting. Clin Exp Dermatol. (2021) 46:1310–1. doi: 10.1111/CED.14688 33864422

[15] Xiong M Pfau J Young AT Wei ML. Artificial intelligence in Teledermatology. Curr Dermatol Rep. (2019) 8:85–90. doi: 10.1007/s13671-019-0259-8

[16] Chin YPH Hou ZY Lee MY Chu HM Wang HH Lin YT. A patient-oriented, general-practitioner-level, deep-learning-based cutaneous pigmented lesion risk classifier on a smartphone. Br J Dermatol. (2020) 182:1498–500. doi: 10.1111/bjd.18859 31907926

[17] Navarro F Escudero-Vinolo M Bescos J. Accurate segmentation and registration of skin lesion images to evaluate lesion change. IEEE J Biomed Health Inform. (2019) 23:501–8. doi: 10.1109/JBHI.2018.2825251 29993849

[18] Webster DE Suver C Doerr M Mounts E Domenico L Petrie T. The mole mapper study, mobile phone skin imaging and melanoma risk data collected using ResearchKit. Sci Data. (2017) 4:1–8. doi: 10.1038/sdata.2017.5 PMC5308198 28195576

[19] Kong FW Horsham C Ngoo A Soyer HP Janda M. Review of smartphone mobile applications for skin cancer detection: what are the changes in availability, functionality, and costs to users over time? Int J Dermatol. (2021) 60:289–308. doi: 10.1111/IJD.15132 32880938

[20] Freeman K Dinnes J Chuchu N Takwoingi Y Bayliss SE Matin RN. Algorithm based smartphone apps to assess risk of skin cancer in adults: systematic review of diagnostic accuracy studies. BMJ. (2020) 368:m127. doi: 10.1136/bmj.m127 32041693 PMC7190019

[21] Matin RN Dinnes J. AI-based smartphone apps for risk assessment of skin cancer need more evaluation and better regulation. Br J Cancer. (2021) 124:1749–50. doi: 10.1038/s41416-021-01302-3 33742148 PMC8144419

[22] Jahn AS Navarini AA Cerminara SE Kostner L Huber SM Kunz M. Over-detection of melanoma-suspect lesions by a CE-certified smartphone app: performance in comparison to dermatologists, 2D and 3D convolutional neural networks in a prospective data set of 1204 pigmented skin lesions involving patients’ perception. Cancers (Basel). (2022) 14:3829. doi: 10.3390/cancers14153829 35954491 PMC9367531

[23] Jones OT Matin RN van der Schaar M Prathivadi Bhayankaram K Ranmuthu CKI Islam MS. Artificial intelligence and machine learning algorithms for early detection of skin cancer in community and primary care settings: a systematic review. Lancet Digit Health. (2022) 4:e466–76. doi: 10.1016/S2589-7500(22)00023-1 35623799

[24] Vuong K Armstrong BK Weiderpass E Lund E Adami H-O Veierod MB. Development and external validation of a melanoma risk prediction model based on self-assessed risk factors. JAMA Dermatol. (2016) 152:889–96. doi: 10.1001/JAMADERMATOL.2016.0939 27276088

[25] Vuong K Armstrong BK Drummond M Hopper JL Barrett JH Davies JR. Development and external validation study of a melanoma risk prediction model incorporating clinically assessed naevi and solar lentigines. Br J Dermatol. (2020) 182:1262–8. doi: 10.1111/BJD.18411 31378928 PMC6997040

[26] Olsen CM Pandeya N Thompson BS Dusingize JC Webb PM Green AC. Risk stratification for melanoma: models derived and validated in a purpose-designed prospective cohort. J Natl Cancer Inst. (2018) 110:1075–83. doi: 10.1093/jnci/djy023, PMID: 29538697

[27] Fontanillas P Alipanahi B Furlotte NA Johnson M Wilson CH Pitts SJ. Disease risk scores for skin cancers. Nature. Communications. (2021) 12:1–13. doi: 10.1038/s41467-020-20246-5, PMID: 33420020 PMC7794415

[28] Roffman D Hart G Girardi M Ko CJ Deng J. Predicting non-melanoma skin cancer via a multi-parameterized artificial neural network. Sci Rep. (2018) 8:1–7. doi: 10.1038/s41598-018-19907-9 29374196 PMC5786038

[29] Wang H-H Wang Y-H Liang C-W Li Y-C. Assessment of deep learning using nonimaging information and sequential medical records to develop a prediction model for nonmelanoma skin Cancer. JAMA Dermatol. (2019) 155:1277–83. doi: 10.1001/JAMADERMATOL.2019.2335 31483437 PMC6727683

[30] Huang C-W Nguyen APA Wu C-C Yang H-C Li Y-C. Develop a prediction model for nonmelanoma skin Cancer using deep learning in EHR data. Stud Comput Intellig. (2021) 899:11–8. doi: 10.1007/978-3-030-49536-7_2

[31] Bakshi A Yan M Riaz M Polekhina G Orchard SG Tiller J. Genomic risk score for melanoma in a prospective study of older individuals. JNCI J Natl Cancer Inst. (2021) 113:1379–85. doi: 10.1093/JNCI/DJAB076 33837773 PMC8921762

[32] Kaiser I Pfahlberg AB Uter W Heppt MV Veierød MB Gefeller O. Risk prediction models for melanoma: a systematic review on the heterogeneity in model development and validation. Int J Environ Res Public Health. (2020) 17:7919. doi: 10.3390/IJERPH17217919 33126677 PMC7662952

[33] Sies K Winkler JK Fink C Bardehle F Toberer F Buhl T. Past and present of computer-assisted dermoscopic diagnosis: performance of a conventional image analyser versus a convolutional neural network in a prospective data set of 1,981 skin lesions. Eur J Cancer. (2020) 135:39–46. doi: 10.1016/j.ejca.2020.04.043 32534243

[34] Abhishek K Kawahara J Hamarneh G. Predicting the clinical management of skin lesions using deep learning. Sci Rep. (2021) 11:7769–14. doi: 10.1038/s41598-021-87064-7, PMID: 33833293 PMC8032721

[35] Winkler JK Blum A Kommoss K Enk A Toberer F Rosenberger A. Assessment of diagnostic performance of dermatologists cooperating with a convolutional neural network in a prospective clinical study: human with machine. JAMA Dermatol. (2023) 159:621–7. doi: 10.1001/jamadermatol.2023.0905 37133847 PMC10157508

[36] Winkler JK Tschandl P Toberer F Sies K Fink C Enk A. Monitoring patients at risk for melanoma: May convolutional neural networks replace the strategy of sequential digital dermoscopy? Eur J Cancer. (2022) 160:180–8. doi: 10.1016/j.ejca.2021.10.030 34840028

[37] Young AT Vora NB Cortez J Tam A Yeniay Y Afifi L. The role of technology in melanoma screening and diagnosis. Pigm Cell Melanoma Res. (2020) 34:288–300. doi: 10.1111/pcmr.12907 32558281

[38] Bozkurt A Kose K Coll-Font J Alessi-Fox C Brooks DH Dy JG. Skin strata delineation in reflectance confocal microscopy images using recurrent convolutional networks with attention. Sci Rep. (2021) 11:12576–11. doi: 10.1038/s41598-021-90328-x, PMID: 34131165 PMC8206415

[39] Mehrabi JN Baugh EG Fast A Lentsch G Balu M Lee BA. A clinical perspective on the automated analysis of reflectance confocal microscopy in dermatology. Lasers Surg Med. (2021) 53:1011–9. doi: 10.1002/LSM.23376 33476062 PMC12551406

[40] Benjamens S Dhunnoo P Meskó B. The state of artificial intelligence-based FDA-approved medical devices and algorithms: an online database. NPJ Digit Med. (2020) 3:118. doi: 10.1038/s41746-020-00324-0 32984550 PMC7486909

[41] The Medical Futurist FDA-approved A.I.-based algorithms. (2022). Available at: https://medicalfuturist.com/fda-approved-ai-based-algorithms/ (Accessed November 7, 2022)

[42] Haenssle HA Fink C Toberer F Winkler J Stolz W Deinlein T. Man against machine reloaded: performance of a market-approved convolutional neural network in classifying a broad spectrum of skin lesions in comparison with 96 dermatologists working under less artificial conditions. Ann Oncol. (2020) 31:137–43. doi: 10.1016/j.annonc.2019.10.013 31912788

[43] MacLellan AN Price EL Publicover-Brouwer P Matheson K Ly TY Pasternak S. The use of noninvasive imaging techniques in the diagnosis of melanoma: a prospective diagnostic accuracy study. J Am Acad Dermatol. (2021) 85:353–9. doi: 10.1016/j.jaad.2020.04.019 32289389

[44] Han SS Kim YJ Moon IJ Jung JM Lee MY Lee WJ. Evaluation of artificial intelligence–assisted diagnosis of skin neoplasms: a single-center, paralleled, unmasked, randomized controlled trial. J Invest Dermatol. (2022) 142:2353–2362.E2. doi: 10.1016/j.jid.2022.02.003 35183551

[45] Onega T Barnhill RL Piepkorn MW Longton GM Elder DE Weinstock MA. Accuracy of digital pathologic analysis vs traditional microscopy in the interpretation of melanocytic lesions. JAMA Dermatol. (2018) 154:1159. doi: 10.1001/jamadermatol.2018.2388 30140929 PMC6233746

[46] Hekler A Utikal JS Enk AH Berking C Klode J Schadendorf D. Pathologist-level classification of histopathological melanoma images with deep neural networks. Eur J Cancer. (2019) 115:79–83. doi: 10.1016/J.EJCA.2019.04.021 31129383

[47] F de Logu Ugolini F Maio V Simi S Cossu A Massi D. Of cutaneous melanoma on digitized histopathological slides via artificial intelligence algorithm. Front Oncol. (2020) 10:1559. doi: 10.3389/FONC.2020.01559, PMID: 33014803 PMC7508308

[48] Wang L Ding L Liu Z Sun L Chen L Jia R. Automated identification of malignancy in whole-slide pathological images: identification of eyelid malignant melanoma in gigapixel pathological slides using deep learning. Br J Ophthalmol. (2020) 104:318–23. doi: 10.1136/bjophthalmol-2018-313706 31302629

[49] Ba W Wang R Yin G Song Z Zou J Zhong C. Diagnostic assessment of deep learning for melanocytic lesions using whole-slide pathological images. Transl Oncol. (2021) 14:101161. doi: 10.1016/J.TRANON.2021.101161 34192650 PMC8254118

[50] del Amor R Launet L Colomer A Moscardó A Mosquera-Zamudio A Monteagudo C. An attention-based weakly supervised framework for Spitzoid melanocytic lesion diagnosis in WSI. Artif Intell Med. (2021) 121:102197. doi: 10.1016/j.artmed.2021.102197 34763799

[51] Olsson H Kartasalo K Mulliqi N Capuccini M Ruusuvuori P Samaratunga H. Estimating diagnostic uncertainty in artificial intelligence assisted pathology using conformal prediction. Nat Commun. (2022) 13:7761. doi: 10.1038/s41467-022-34945-8 36522311 PMC9755280

[52] van Zon MCM van der Waa JD Veta M Krekels GAM. Whole-slide margin control through deep learning in Mohs micrographic surgery for basal cell carcinoma. Exp Dermatol. (2021) 30:733–8. doi: 10.1111/EXD.14306, PMID: 33656186

[53] Kimeswenger S Tschandl P Noack P Hofmarcher M Rumetshofer E Kindermann H. Artificial neural networks and pathologists recognize basal cell carcinomas based on different histological patterns. Mod Pathol. (2020) 34:895–903. doi: 10.1038/s41379-020-00712-7 33184470

[54] Duschner N Baguer DO Schmidt M Griewank KG Hadaschik E Hetzer S. Applying an artificial intelligence deep learning approach to routine dermatopathological diagnosis of basal cell carcinoma. J Dtsch Dermatol Ges. (2023) 21:1329–37. doi: 10.1111/DDG.15180 37814387

[55] Brinker TJ Kiehl L Schmitt M Jutzi TB Krieghoff-Henning EI Krahl D. Deep learning approach to predict sentinel lymph node status directly from routine histology of primary melanoma tumours. Eur J Cancer. (2021) 154:227–34. doi: 10.1016/J.EJCA.2021.05.026 34298373

[56] Kulkarni PM Robinson EJ Pradhan JS Gartrell-Corrado RD Rohr BR Trager MH. Deep learning based on standard H&E images of primary melanoma tumors identifies patients at risk for visceral recurrence and death. Clin Cancer Res. (2020) 26:1126–34. doi: 10.1158/1078-0432.CCR-19-1495 31636101 PMC8142811

[57] Polesie S McKee PH Gardner JM Gillstedt M Siarov J Neittaanmäki N. Attitudes toward artificial intelligence within dermatopathology: an international online survey. Front Med. (2020) 7:591952. doi: 10.3389/FMED.2020.591952 PMC7606983 33195357

[58] Johansson E Månefjord F (2021). Segmentation and prediction of mutation status of malignant melanoma whole-slide images using deep learning.

[59] Coudray N Ocampo PS Sakellaropoulos T Narula N Snuderl M Fenyö D. Classification and mutation prediction from non–small cell lung cancer histopathology images using deep learning. Nat Med. (2018) 24:1559–67. doi: 10.1038/s41591-018-0177-5 30224757 PMC9847512

[60] Fu Y Jung AW Torne RV Gonzalez S Vöhringer H Shmatko A. Pan-cancer computational histopathology reveals mutations, tumor composition and prognosis. Nat Can. (2020) 1:800–10. doi: 10.1038/s43018-020-0085-8 35122049

[61] Kather JN Heij LR Grabsch HI Loeffler C Echle A Muti HS. Pan-cancer image-based detection of clinically actionable genetic alterations. Nat Can. (2020) 1:789–99. doi: 10.1038/s43018-020-0087-6 PMC7610412 33763651

[62] Torres R Judson-Torres RL. Research techniques made simple: feature selection for biomarker discovery. J Invest Dermatol. (2019) 139:2068–2074.e1. doi: 10.1016/j.jid.2019.07.682 31543209

[63] Gerami P Yao Z Polsky D Jansen B Busam K Ho J. Development and validation of a noninvasive 2-gene molecular assay for cutaneous melanoma. J Am Acad Dermatol. (2017) 76:114–120.e2. doi: 10.1016/J.JAAD.2016.07.038 27707590 PMC5599145

[64] Jackson SR Jansen B Yao Z Ferris LK. Risk stratification of severely dysplastic nevi by non-invasively obtained gene expression and mutation analyses. SKIN J Cutan Med. (2020) 4:124–9. doi: 10.25251/skin.4.2.5

[65] Ludzik J Lee C Witkowski A. Potential limitations in the clinical adoption of 3-GEP pigmented lesion assay for melanoma triage by dermatologists and advanced practice practitioners. Cureus. (2022) 14:e31914. doi: 10.7759/cureus.31914 36579219 PMC9792410

[66] Estrada S Shackelton J Cleaver N Depcik-Smith N Cockerell C Lencioni S. Development and validation of a diagnostic 35-gene expression profile test for ambiguous or difficult-to-diagnose suspicious pigmented skin lesions. SKIN J Cutan Med. (2020) 4:506–22. doi: 10.25251/skin.4.6.3

[67] Torres R Lang UE Hejna M Shelton SJ Joseph NM Shain AH. MicroRNA ratios distinguish melanomas from nevi. J Invest Dermatol. (2019) 140:164–173.E7. doi: 10.1016/j.jid.2019.06.126 31580842 PMC6926155

[68] Grossman D Okwundu N Bartlett EK Marchetti MA Othus M Coit DG. Prognostic gene expression profiling in cutaneous melanoma: identifying the knowledge gaps and assessing the clinical benefit. JAMA Dermatol. (2020) 156:1004–11. doi: 10.1001/JAMADERMATOL.2020.1729 32725204 PMC8275355

[69] Swetter SM Thompson JA Albertini MR Barker CA Baumgartner J Boland G. NCCN guidelines® insights: melanoma: cutaneous, version 2.2021: featured updates to the NCCN guidelines. J Natl Compr Cancer Netw. (2021) 19:364–76. doi: 10.6004/JNCCN.2021.0018 33845460

[70] Jarell A Gastman BR Dillon LD Hsueh EC Podlipnik S Covington KR. Optimizing treatment approaches for patients with cutaneous melanoma by integrating clinical and pathologic features with the 31-gene expression profile test. J Am Acad Dermatol. (2022) 87:1312–20. doi: 10.1016/J.JAAD.2022.06.1202 35810840

[71] Varedi A Gardner LJ Kim CC Chu EY Ming ME Leachman SA. Use of new molecular tests for melanoma by pigmented-lesion experts. J Am Acad Dermatol. (2020) 82:245–7. doi: 10.1016/J.JAAD.2019.08.022 31415835

[72] Kashani-Sabet M Leachman SA Stein JA Arbiser JL Berry EG Celebi JT. Early detection and prognostic assessment of cutaneous melanoma. JAMA Dermatol. (2023) 159:545–53. doi: 10.1001/jamadermatol.2023.0127 36920356 PMC11225588

[73] Jones K Lennon E McCathie K Millar A Isles C McFadyen A. Teledermatology to reduce face-to-face appointments in general practice during the COVID-19 pandemic: a quality improvement project. BMJ Open Qual. (2022) 11:e001789. doi: 10.1136/BMJOQ-2021-001789 PMC9136693 35618315

[74] Winkler JK Sies K Fink C Toberer F Enk A Abassi MS. Association between different scale bars in dermoscopic images and diagnostic performance of a market-approved deep learning convolutional neural network for melanoma recognition. Eur J Cancer. (2021) 145:146–54. doi: 10.1016/J.EJCA.2020.12.010 33465706

[75] Winkler JK Fink C Toberer F Enk A Deinlein T Hofmann-Wellenhof R. Association between surgical skin markings in Dermoscopic images and diagnostic performance of a deep learning convolutional neural network for melanoma recognition. JAMA Dermatol. (2019) 155:1135–41. doi: 10.1001/JAMADERMATOL.2019.1735 31411641 PMC6694463

[76] Katragadda C Finnane A Soyer HP Marghoob AA Halpern A Malvehy J. Technique standards for skin lesion imaging: a Delphi consensus statement. JAMA Dermatol. (2017) 153:207–13. doi: 10.1001/JAMADERMATOL.2016.3949 27892996

[77] Daneshjou R Barata C Betz-Stablein B Celebi ME Codella N Combalia M. Checklist for evaluation of image-based artificial intelligence reports in dermatology: CLEAR Derm consensus guidelines from the international skin imaging collaboration artificial intelligence working group. JAMA Dermatol. (2022) 158:90–6. doi: 10.1001/JAMADERMATOL.2021.4915 34851366 PMC9845064

[78] Lester JC Clark L Linos E Daneshjou R. Clinical photography in skin of colour: tips and best practices. Br J Dermatol. (2021) 184:1177–9. doi: 10.1111/BJD.19811 33448346

[79] Jae HK Soo HS Young CK Hyo HA. The influence of photography education on quality of medical photographs taken by dermatology resident. Kor J Dermatol. (2008) 46:1042–7.

[80] Sun MD Kentley J Wilson BW Soyer HP Curiel-Lewandrowski CN Rotemberg V. Digital skin imaging applications, part I: assessment of image acquisition technique features. Skin Res Technol. (2022) 28:623–32. doi: 10.1111/SRT.13163 35652379 PMC9907654

[81] Vodrahalli K Daneshjou R Novoa RA Chiou A Ko JM Zou J. TrueImage: a machine learning algorithm to improve the quality of telehealth photos. Pac Symp Biocomput. (2021) 26:220–31. doi: 10.1142/9789811232701_0021 33691019

[82] Chen RJ Wang JJ Williamson DFK Chen TY Lipkova J Lu MY. Algorithm fairness in artificial intelligence for medicine and healthcare. Nat Biomed Eng. (2023) 7:719. doi: 10.1038/S41551-023-01056-8 37380750 PMC10632090

[83] Daneshjou R Vodrahalli K Novoa RA Jenkins M Liang W Rotemberg V. Disparities in dermatology AI performance on a diverse, curated clinical image set. Sci Adv. (2022) 8:6147. doi: 10.1126/SCIADV.ABQ6147/SUPPL_FILE/SCIADV.ABQ6147_SM.PDF PMC9374341 35960806

[84] ISIC (2018). ISIC-Archive. Available at: https://www.isic-archive.com/#!/topWithHeader/wideContentTop/main (Accessed November 1, 2018).

[85] Daneshjou R Smith MP Sun MD Rotemberg V Zou J. Lack of transparency and potential Bias in artificial intelligence data sets and algorithms: a scoping review. JAMA Dermatol. (2021) 157:1362–9. doi: 10.1001/JAMADERMATOL.2021.3129 34550305 PMC9379852

[86] Han SS Moon IJ Kim SH Na J-I Kim MS Park GH. Assessment of deep neural networks for the diagnosis of benign and malignant skin neoplasms in comparison with dermatologists: a retrospective validation study. PLoS Med. (2020) 17:e1003381. doi: 10.1371/JOURNAL.PMED.1003381 33237903 PMC7688128

[87] Han SS Kim MS Lim W Park GH Park I Chang SE. Classification of the clinical images for benign and malignant cutaneous tumors using a deep learning algorithm. J Invest Dermatol. (2018) 138:1529–38. doi: 10.1016/j.jid.2018.01.028 29428356

[88] Brinker TJ Hekler A Hauschild A Berking C Schilling B Enk AH. Comparing artificial intelligence algorithms to 157 German dermatologists: the melanoma classification benchmark. Eur J Cancer. (2019) 111:30–7. doi: 10.1016/J.EJCA.2018.12.016 30802784

[89] Van Beek MJ Swerlick RA Mathes B Hruza GJ Resneck J Pak HS. The 2020 annual report of DataDerm: the database of the American Academy of Dermatology. J Am Acad Dermatol. (2021) 84:1037–41. doi: 10.1016/j.jaad.2020.11.068 33316331

[90] Finlayson SG Bowers JD Ito J Zittrain JL Beam AL Kohane IS. Adversarial attacks on medical machine learning. Science. (2019) 363:1287–9. doi: 10.1126/science.aaw4399 30898923 PMC7657648

[91] Navarrete-Dechent C Liopyris K Marchetti MA. Multiclass artificial intelligence in dermatology: Progress but still room for improvement. J Invest Dermatol. (2021) 141:1325–8. doi: 10.1016/J.JID.2020.06.040 33049269 PMC9275671

[92] Lee G-H Ko H-B Lee S-W (2021). Joint dermatological lesion classification and confidence modeling with uncertainty estimation. arXiv [Preprint]. doi: 10.48550/arXiv.2107.08770

[93] Kovarik C Lee I Ko J Adamson A Otley C Kvedar J. Commentary: position statement on augmented intelligence (AuI). J Am Acad Dermatol. (2019) 81:998–1000. doi: 10.1016/j.jaad.2019.06.032 31247221

[94] Cortez JL Vasquez J Wei ML. The impact of demographics, socioeconomics, and health care access on melanoma outcomes. J Am Acad Dermatol. (2021) 84:1677–83. doi: 10.1016/J.JAAD.2020.07.125 32783908

[95] Feng H Berk-Krauss J Feng PW Stein JA. Comparison of dermatologist density between urban and rural counties in the United States. JAMA Dermatol. (2018) 154:1265–71. doi: 10.1001/jamadermatol.2018.3022 30193349 PMC6248119

[96] Ashrafzadeh S Nambudiri VE. The COVID-19 Crisis: A Unique Opportunity to Expand Dermatology to Underserved Populations Mosby Inc. J Am Acad Dermatol. (2020) 83:e83–e84. doi: 10.1016/j.jaad.2020.04.154 32380217 PMC7198179

[97] Minagawa A Koga H Sano T Matsunaga K Teshima Y Hamada A. Dermoscopic diagnostic performance of Japanese dermatologists for skin tumors differs by patient origin: a deep learning convolutional neural network closes the gap. J Dermatol. (2021) 48:232–6. doi: 10.1111/1346-8138.15640 33063398

[98] Tschandl P Rinner C Apalla Z Argenziano G Codella N Halpern A. Human–computer collaboration for skin cancer recognition. Nat Med. (2020) 26:1229–34. doi: 10.1038/s41591-020-0942-0 32572267

[99] Winkler JK Sies K Fink C Toberer F Enk A Abassi MS. Collective human intelligence outperforms artificial intelligence in a skin lesion classification task. J Dtsch Dermatol Ges. (2021) 19:1178–84. doi: 10.1111/DDG.14510 34096688

[100] Felmingham CM Adler NR Ge Z Morton RL Janda M Mar VJ. The importance of incorporating human factors in the design and implementation of artificial intelligence for skin Cancer diagnosis in the real world. Am J Clin Dermatol. (2020) 22:233–42. doi: 10.1007/S40257-020-00574-4 33354741

[101] Gaube S Suresh H Raue M Merritt A Berkowitz SJ Lermer E. Do as AI say: susceptibility in deployment of clinical decision-aids. NPJ Digit Med. (2021) 4:1–8. doi: 10.1038/s41746-021-00385-9 33608629 PMC7896064

[102] Sheller MJ Edwards B Reina GA Martin J Pati S Kotrotsou A. Federated learning in medicine: facilitating multi-institutional collaborations without sharing patient data. Sci Rep. (2020) 10:12598. doi: 10.1038/S41598-020-69250-1 32724046 PMC7387485

[103] McMahan B Moore E Ramage D Hampson S Arcas BAY (2017). Communication-efficient learning of deep networks from decentralized data. arXiv [Preprint]. doi: 10.48550/arXiv.1602.05629

[104] Zhang DY Kou Z Wang D (2020). “FairFL: a fair federated learning approach to reducing demographic bias in privacy-sensitive classification models” in *Proceedings—2020 IEEE International Conference on Big Data, Big Data 2020*.

[105] Lipkova J Chen RJ Chen B Lu MY Barbieri M Shao D. Artificial intelligence for multimodal data integration in oncology. Cancer Cell. (2022) 40:1095. doi: 10.1016/J.CCELL.2022.09.012 36220072 PMC10655164

[106] Yap J Yolland W Tschandl P. Multimodal skin lesion classification using deep learning. Exp Dermatol. (2018) 27:1261–7. doi: 10.1111/EXD.13777 30187575

[107] Berkowitz SJ Kwan D Cornish TC Silver EL Thullner KS Aisen A. Interactive multimedia reporting technical considerations: HIMSS-SIIM collaborative white paper. J Digit Imaging. (2022) 35:817–33. doi: 10.1007/S10278-022-00658-Z 35962150 PMC9485305

[108] Morgado AC Andrade C Teixeira LF Vasconcelos MJM. Incremental learning for dermatological imaging modality classification. J Imaging. (2021) 7:180. doi: 10.3390/JIMAGING7090180 34564106 PMC8469804

[109] Gottumukkala VSSPR Kumaran N Sekhar VC. BLSNet: skin lesion detection and classification using broad learning system with incremental learning algorithm. Expert Syst. (2022) 39:e12938. doi: 10.1111/exsy.12938

[110] Bissoto A Perez F Valle E Avila S. Skin lesion synthesis with generative adversarial networks. Lect Notes Comput Sci. (2018) 11041. doi: 10.1007/978-3-030-01201-4_32

[111] Carrasco Limeros S Majchrowska S Zoubi MK Rosén A Suvilehto J Sjöblom L. (2023). “Assessing GAN-Based Generative Modeling on Skin Lesions Images” in *MIDI 2022: Digital Interaction and Machine Intelligence*. pp. 93–102. doi: 10.1007/978-3-031-37649-8_10

[112] Salvi M Branciforti F Veronese F Zavattaro E Tarantino V Savoia P. DermoCC-GAN: a new approach for standardizing dermatological images using generative adversarial networks. Comput Methods Prog Biomed. (2022) 225:107040. doi: 10.1016/j.cmpb.2022.107040 35932723

[113] Esteva A Chou K Yeung S Naik N Madani A Mottaghi A. Deep learning-enabled medical computer vision. NPJ Digit Med. (2021) 4:5. doi: 10.1038/s41746-020-00376-2 33420381 PMC7794558

[114] Gu J Wang Z Kuen J Ma L Shahroudy A Shuai B. Recent advances in convolutional neural networks. Pattern Recogn. (2018) 77:354–77. doi: 10.1016/J.PATCOG.2017.10.013

[115] Shamshad F Khan S Zamir SW Khan MH Hayat M Khan FS. Transformers in medical imaging: a survey. Med Image Anal. (2023) 88:102802. doi: 10.1016/j.media.2023.102802 37315483

[116] Khan S Ali H Shah Z. Identifying the role of vision transformer for skin cancer—a scoping review. Front Artif Intell. (2023) 6:1202990. doi: 10.3389/FRAI.2023.1202990/BIBTEX 37529760 PMC10388102

[117] Liu Z Mao H Wu C-Y Feichtenhofer C Darrell T Xie S. A ConvNet for the 2020s. Available at: https://github.com/facebookresearch/ConvNeXt (Accessed February 22, 2024).

[118] Zhou J He X Sun L Xu J Chen X Chu Y. (2023). Pre-trained multimodal large language model enhances dermatological diagnosis using SkinGPT-4. medRxiv [Preprint]. doi: 10.1101/2023.06.10.23291127 PMC11226626 38969632

[119] Krishna K Khosla S Bigham J Lipton ZC (2021). “Generating SOAP notes from doctor-patient conversations using modular summarization techniques” in *ACL-IJCNLP 2021—59th Annual Meeting of the Association for Computational Linguistics and the 11th International Joint Conference on Natural Language Processing, Proceedings of the Conference*. 4958–4972.

[120] May A.I. (2023). Someday Work Medical Miracles. For Now, It Helps Do Paperwork. The New York Times. Available at: https://www.nytimes.com/2023/06/26/technology/ai-health-care-documentation.html (Accessed February 22, 2024).

[121] Matin RN Linos E Rajan N. Leveraging large language models in dermatology. Br J Dermatol. (2023) 189:253–4. doi: 10.1093/BJD/LJAD230 37410567

[122] Omiye JA Lester JC Spichak S Rotemberg V Daneshjou R. Large language models propagate race-based medicine. NPJ Digit Med. (2023) 6:195. doi: 10.1038/s41746-023-00939-z 37864012 PMC10589311

[123] Deng J Dong W Socher R Li L-J Li K Fei-Fei L (2010). “ImageNet: A large-scale hierarchical image database” in *2009 IEEE Conference on Computer Vision and Pattern Recognition*. 248–255.

[124] Azizi S Culp L Freyberg J Mustafa B Baur S Kornblith S. Robust and data-efficient generalization of self-supervised machine learning for diagnostic imaging. Nat Biomed Eng. (2023) 7:756–79. doi: 10.1038/s41551-023-01049-7 37291435

